# Effect of Modulated Alternating and Direct Current Iontophoresis on Transdermal Delivery of Lidocaine Hydrochloride

**DOI:** 10.1155/2014/537941

**Published:** 2014-05-15

**Authors:** Gaurav Bhatia, Ajay K. Banga

**Affiliations:** Department of Pharmaceutical Sciences, College of Pharmacy, Mercer University, Atlanta, GA 30341, USA

## Abstract

The objective of this study was to investigate the iontophoretic delivery of lidocaine hydrochloride through porcine skin and to compare the effects of modulated alternating and direct current iontophoresis. Continuous and modulated iontophoresis was applied for one hour and two hours (0-1 h and 4-5th h) using a 1% w/v solution of lidocaine hydrochloride. Tape stripping was done to quantify the amount of drug permeated into stratum corneum and skin extraction studies were performed to determine the amount of drug in stripped skin. Receptor was sampled and analyzed over predefined time periods. The amount of lidocaine delivered across porcine skin after modulated direct current iontophoresis for 2 h was 1069.87 ± 120.03 **μ**g/sq*·*cm compared to 744.81 ± 125.41 **μ**g/sq*·*cm after modulated alternating current iontophoresis for 2 h. Modulated direct current iontophoresis also enhanced lidocaine delivery by twelvefold compared to passive delivery as 91.27 ± 18.71 **μ**g/sq*·*cm of lidocaine was delivered after passive delivery. Modulated iontophoresis enhanced the delivery of lidocaine hydrochloride across porcine skin compared to the passive delivery. Modulated alternating current iontophoresis for duration of 2 h at frequency of 1 kHz was found to be comparable to the continuous direct current iontophoresis for 1 h.

## 1. Introduction

Lidocaine hydrochloride is a hydrophilic local anesthetic, which is widely used for topical anesthesia and other medical and surgical procedures including treatment of skin sores, lesions, and suturing of wounds [1]. It is also used as an antiarrhythmic drug [2]. It exerts local anesthetic effect by binding with voltage gated Na^+^ channels at axonal membrane and prevents the transport of Na^+^ across the channels, thus inhibiting the postsynaptic neuron from depolarization and stabilizes neuronal membrane [3]. The most common form of lidocaine administration is through intravenous or hypodermic injection, which causes pain and discomfort [4]. Transdermal delivery of lidocaine is a potential alternative route of administration.

However, due to poor penetration through intact skin, the percutaneous application of lidocaine is limited [5]. Commercial products including EMLA cream (AstraZeneca) and Lidoderm (Endo Laboratories) are available for transdermal delivery of lidocaine. However, achieving effective analgesia requires the application of EMLA for 1-2 h, which limits its use during emergency where fast onset of anesthesia is desired making it less convenient to use during normal clinical procedures [6]. Several other formulations such as liposomes [5] or microemulsions have also been investigated to enhance the transdermal delivery. Polymeric liposomes have been shown to be effective in enhancing the transdermal delivery of lidocaine across the mouse skin. Bacterial cellulose membrane incorporated with lidocaine demonstrated lower permeation than conventional formulation through human epidermis [7]. A combination of short-term iontophoresis and microemulsion formulation significantly increased the flux and resulted in accumulation of large skin drug depot and short lag time in delivery of lidocaine through porcine skin. Studies have also reported that transdermal delivery of lidocaine has a possibility to be used for local anesthesia and pain management of the skin [8]. Therefore, there is a need to enhance the transdermal delivery of lidocaine to achieve rapid onset of action; this can be achieved using physical enhancement techniques such as iontophoresis.

Iontophoresis is a widely used technique for the delivery of neutral and charged drug molecules into and across the skin by using small amount of physiological current [9]. The mechanisms of iontophoresis include electrorepulsion, which is based on the principle of “like repels like” and electroosmosis where the neutral molecules are transported from anode to cathode along with the bulk solvent flow. Direct current (DC) iontophoresis is the most commonly used form of the transdermal iontophoretic drug delivery. Examples of drug delivery using DC iontophoresis are the Numby Stuff Phoresor system (Iomed, Inc., UT), LidoSite (lidocaine hydrochloride/epinephrine topical iontophoretic patch), and the Ionsys E-Trans system for systemic fentanyl delivery (Alza Corp., CA). However, DC iontophoresis may have some adverse effects including electrical burn as a result of electrode polarization during electrolysis. This adverse effect limits the duration time of DC iontophoresis to less than 15 min at current density of 1 mA/cm^2^ [10]. A decrease in transport efficiency is also observed in DC iontophoresis with increasing duration of the electric application. The decrease transport efficiency is due to the voltage drop in the solution, which results from the formation of an electric double layer on the surface of electrode known as electrode polarization; this phenomenon occurs due to the accumulation of ionized substance with the different charge from that of the electrode. To overcome these issues, alternating current (AC) has also been employed in iontophoretic delivery [11]. It has been reported that AC iontophoresis can eliminate electrochemical burn and reduce the skin irritation, which occurs during the long application time of DC iontophoresis [12]. It has also been reported that alternating current iontophoresis can reduce the skin electrical resistance, thereby increasing the intrinsic permeability of skin [11].

Iontophoresis is also widely used to enhance the delivery of topical anesthetics [13]. Studies have also reported that iontophoresis facilitates the transport of lidocaine molecules into the skin under the influence of electric current and can provide topical anesthesia of intact skin within 5–15 min [14]. Lidocaine iontophoresis has also been found to be effective in reducing the pain associated with venous cannulation in patients [15].

The objective of the current study was to enhance the transdermal delivery of lidocaine by iontophoresis and to compare the effects of modulated alternating and direct current iontophoresis on the permeation of lidocaine in porcine full thickness skin. Passive diffusion of lidocaine was used as the control for the study.

## 2. Materials and Methods

### 2.1. Materials

Lidocaine hydrochloride, silver wire (0.5 mm diameter), and silver chloride used for preparation of electrodes were purchased from Sigma-Aldrich (St. Louis, MO, USA). Acetonitrile, methanol, potassium phosphate monobasic (KH_2_PO_4_), and PBS (phosphate buffered saline) were purchased from Fisher Scientific (NJ, USA). Transpore tape for tape stripping was obtained from 3 M (St. Paul, MN, USA). Deionized water was used to prepare all the solutions required in this study and for HPLC analysis. Iontophoresis power supply unit (Model 6221) was purchased from Keithley Instruments (Cleveland, OH, USA).

### 2.2. Methods

#### 2.2.1. Skin Isolation and Preparation

Porcine skin was obtained from a local abattoir. Whole skin was excised followed by the removal of subcutaneous fat. The skin was then cleaned using deionized water and stored at −20°C in aluminum foil until use. Skin was thawed before permeation studies, cut into appropriate sizes, and mounted on the Franz diffusion cells (PermeGear, Hellertown, PA, USA) with stratum corneum side facing the donor compartment and secured into place using clamps.

#### 2.2.2. Preparation of Electrodes

A planar coil of silver was prepared manually and used as the anode in the study. The cathode was custom made by coating a melt of silver chloride on silver wire. Coating was done until a uniform and sufficient coat of the silver chloride was obtained.

#### 2.2.3. Continuous versus Modulated Iontophoresis

Continuous and modulated iontophoresis were applied for the duration of one and two hours. The anode was placed in the donor chamber and the cathode was inserted into the receptor compartment through the sampling arm to perform anodal iontophoresis. A continuous direct current (DC) iontophoresis at current density of 0.5 mA/cm^2^ and alternating current (AC) iontophoresis at a frequency of 1 kHz and current density of 0.5 mA/cm^2^ were applied using Keithley instrument (Model 6221; Cleveland, OH, USA) for one hour from 0 to 1 h.

To determine the effect of iontophoresis on transdermal delivery of lidocaine, continuous, direct current iontophoresis was applied for one hour from 0 to 1 h and modulated direct current iontophoresis at current density of 0.5 mA/cm^2^ was applied for two hours (from 0 to 1 h and from 4 to 5th h), while modulated alternating current iontophoresis at a frequency of 1 kHz and current density of 0.5 mA/cm^2^ was also applied for two hours (from 0 to 1 h and from 4 to 5th h), respectively, on porcine full thickness skin. Our group has also reported that flux recovers back to the normal level in two to three hours after iontophoresis [16]. Therefore, we kept interim period of 3 h between two iontophoresis applications. Receptor samples (0.5 mL) were collected at predetermined time intervals during the study.

#### 2.2.4. Permeation Studies

In this study, the influence of anodal iontophoresis on the delivery of lidocaine hydrochloride into porcine full thickness skin was investigated, and passive diffusion was used as the control in the study. In vitro permeation studies (*n* ≥ 3) were performed using vertical Franz diffusion cells. Receptor compartment was thoroughly washed prior to the study and then filled with receptor buffer (5 mL 1X PBS; pH 7.4). The temperature of the cells was maintained at 37°C during the study by using a water circulation jacket built around receptor chamber. Porcine skin was mounted on the receptor compartments (effective area of diffusion was 0.64 cm^2^) with the stratum corneum facing the donor chamber. Donor chambers were then placed on the mounted skin and secured into place using clamps. Lidocaine (1% w/v) solution in deionized water was used as the donor (0.5 mL) for the study. Sodium chloride (23 mM) was added to the donor solution to drive the electrochemistry of the silver-silver chloride electrodes. Samples (0.5 mL) were withdrawn from the receptor compartment at predetermined time intervals over a period of 24 h and replenished with equal volume of fresh receptor buffer. Samples obtained were analyzed using high performance liquid chromatography (HPLC) assay. After the permeation studies, tape stripping and skin extraction studies were performed to quantify drug levels in the stratum corneum and the stripped skin, respectively.

Lidocaine is a small molecule and is categorized pharmacologically as local anesthetic and antiarrhythmic drug. It is lipophilic in base form with a log *P* of 2.6, while the hydrochloride salt of drug (used here) is hydrophilic in nature with log *P* ≤ 0 [17]. The salt form of drug was used for the study as active enhancement techniques like iontophoresis require the drug to be hydrophilic and in charged form for the delivery; also salt form of drug has the ability to provide the chloride ions, which is essential for the completion of electrochemical reaction at anode when silver/silver chloride electrodes are used for iontophoresis [18]. The electrochemistry at anode and cathode is as follows.

 Anode:
  (1) $$\begin{array}{c} \text{Ag}+\text{C}{\text{l}}^{-}⟶\text{AgCl}+{\text{e}}^{-} \end{array}$$
 Cathode:
  (2) $$\begin{array}{c} \text{AgCl}+{\text{e}}^{-}⟶\text{Ag}+\text{C}{\text{l}}^{-} \end{array}$$

For salt form of the drug, log *D* (logarithm of distribution coefficient) is used, which is the partition between organic and buffer phase and is determined by degree of ionization of molecule at a particular pH and pKa [19]. log *D* was calculated by (3) as follows [20].

(3) $$\begin{array}{c} \log D=\log P-\log\left(1+10\wedge \left(\text{pKa}-\text{pH}\right)\right). \end{array}$$

The log *D* value of compound also determines its ability to ionize at given pH condition, so the effectiveness of transport by iontophoresis through electrorepulsion can be determined. Lidocaine hydrochloride has a log *D* of 1.57 (calculated from (3)) at pH 7.4 and the drug has pKa of 7.9; hence, at pH of 7.4, it will be positively charged and anodal iontophoresis will actively transport lidocaine by electrorepulsion.

#### 2.2.5. Skin Extraction

Skin extraction procedure was performed to determine the drug levels in skin. Skin samples were removed from Franz diffusion cells at the end of permeation studies. The skin surface was then thoroughly cleaned by dabbing it three times with Q-tips soaked in receptor medium. Skin was then tape stripped using 3 M Transpore tapes to determine the amount of drug permeated in stratum corneum. The first five tape strips were extracted individually and remaining tape strips were extracted in a group of five. Kim wipes were used to dry the skin surface. After tape stripping, skin samples were minced manually using a pair of scissors and added to scintillation vials. PBS (1X, pH7.4) was used as an extraction solvent and was added to minced skin. The extraction was carried out by shaking the vials overnight on the roller shaker (New Brunswick Scientific Co. Inc, NJ, USA). The samples were then centrifuged at 13400 g for 2 min at 200 rpm and the supernatant extract was filtered by using 0.45 *μ*m filters (Milipore) and analyzed using HPLC assay.

#### 2.2.6. Quantitative Analysis

Lidocaine hydrochloride was quantified using HPLC by using modified assay from literature. HPLC analysis was performed on Perkin Elmer System (Waltham, MA) with a UV detector operating at 230 nm. Column used was RP-18 Phenomenex column (Luna 5 *μ* C18 100A, 250 mm × 4.6 mm, Phenomenex, Torrance, CA, USA). Mobile phase consisted of methanol: 0.1 M sodium dihydrogen phosphate (60 : 40%, v/v). Isocratic elution was performed at a flow rate of 0.6 mL/min after injecting 10 *μ*L of sample, the total run time was 10 min and the retention time of lidocaine hydrochloride was around 6.04 min. The Lower limit of detection (LOD) was 0.05 *μ*g and the lower limit of quantification (LOQ) was 0.1 *μ*g. Standards were prepared in the range of 0.1–100 *μ*g. The assay was sensitive for range of interest.

#### 2.2.7. Statistical Analysis

Statistical significance was determined using one-way analysis of variance (ANOVA) and Dunnett's test using GraphPad Prism software (version 5.0d). All results are reported as mean ± SD (*n* = 3). Values were considered significantly different when *P* ≤ 0.5.

## 3. Results and Discussion

### 3.1. Iontophoretic Drug Transport Mechanism

**Figure 1:**
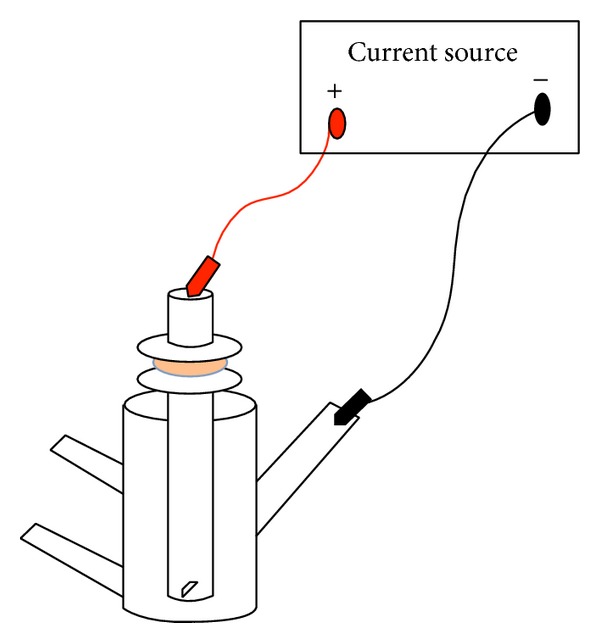
Iontophoretic setup that was used for iontophoretic studies of lidocaine, silver wire (represented in red) served as the anode (active electrode) and silver wire coated with silver chloride (represented in black) serve as cathode. The electrodes were connected to a current source to perform anodal iontophoresis.

Anodal iontophoresis was performed for one or two hours using alternating and direct current (Figure 1) to determine the effect of iontophoresis on the delivery of lidocaine hydrochloride through porcine full thickness skin. The present study revealed that both the DC and AC iontophoreses enhanced the delivery of lidocaine hydrochloride through porcine skin. Studies have demonstrated that the mechanisms responsible for the transport of drug substance after DC iontophoresis are electrorepulsion, electroosmosis, and increased skin permeability [21]. It has been reported that electrorepulsion and electroosmosis are involved in transport of lidocaine after the application of AC iontophoresis at low frequency. Lidocaine used in the study is dissociated into positively charged lidocaine and hydrogen ions along with negatively charged chloride ions. During electrorepulsion the charged substances are repelled from the electrode of same polarity [22]. Positively charge lidocaine ions would be similarly repelled during the positive phase of AC iontophoresis. Transport mechanism of the substance after the application of electric field can be explained by following (4) [10]:

(4) $$\begin{array}{c} J_{\text{L}}=J_{p}+J_{\text{er}}+J_{\text{eo}}, \end{array}$$

where *J*_L_ is the mole flux of the substance L, *J*_*p*_ is the passive flux, *J*_er_ represents electrorepulsive contribution, and *J*_eo_ depicts the electroosmotic flux. Therefore, both electrorepulsion and electroosmosis are the major mechanisms responsible for the delivery of lidocaine hydrochloride after AC and DC iontophoresis.

### 3.2. Effect of Continuous Iontophoresis on Lidocaine Delivery through Porcine Skin

**Figure 2:**
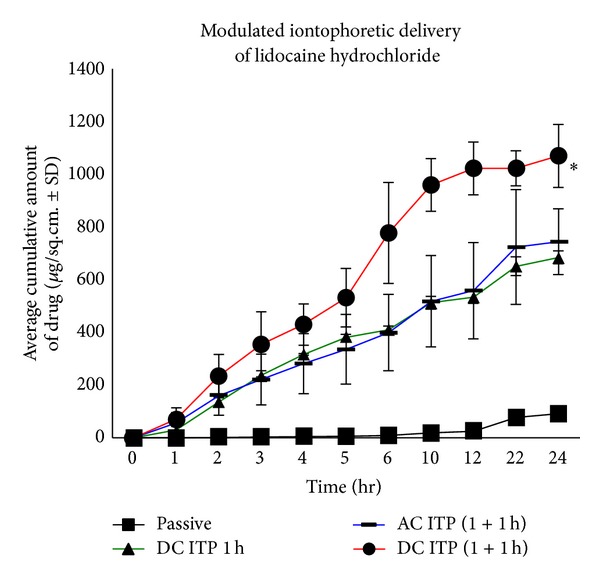
Cumulative amount of lidocaine delivered through full thickness porcine ear skin after continuous and modulated iontophoresis (*P < 0.05 versus passive; mean ± SD, *n* = 3).

**Figure 3:**
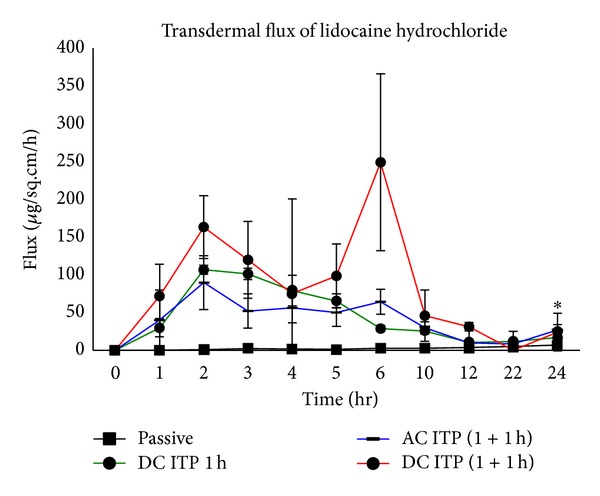
Flux of lidocaine across full thickness pig ear skin after continuous and modulated iontophoresis *P < 0.05 versus passive; mean ± SD, *n* = 3).

Continuous iontophoresis using alternating and direct current iontophoresis enhanced the delivery of lidocaine into porcine full thickness skin as compared to passive diffusion. The amount of drug delivered after continuous DC iontophoresis for one hour was 684.76 ± 24.81 *μ*g/sq·cm compared to 91.27 ± 18.71 *μ*g/cm^2^ for passive delivery (Figure 2). Continuous iontophoresis resulted in a steady rise of drug delivered when the current was applied and the highest flux was 106.59 ± 5.85 *μ*g/cm^2^/h at 2 h for DC iontophoresis after which the flux decreases gradually (Figure 3).

### 3.3. Effect of Modulated Iontophoresis on Lidocaine Delivery through Porcine Skin

To determine the effect of modulated iontophoresis on the delivery of lidocaine modulated anodal iontophoresis (0.5 mA/cm^2^) using direct and alternating current, iontophoresis was performed for two hours (from 0-1 h and 4-5th h) on porcine full thickness skin and passive diffusion served as the control for the study.

Modulated iontophoresis enhanced the delivery of lidocaine significantly (*P* < 0.05) from 91.27 ± 18.71 *μ*g/cm^2^ for passive delivery to 744.81 ± 125.41 *μ*g/cm^2^ for AC iontophoresis and 1069.87 ± 120.01 *μ*g/cm^2^ for DC iontophoresis. The amount of drug delivered by modulated alternating current iontophoresis for duration of two hours at frequency of 1 kHz was found to be comparable to the drug delivered by continuous direct current iontophoresis for one hour as 744.81 ± 125.41 *μ*g/cm^2^ of lidocaine was delivered after modulated AC iontophoresis compared to 684.76 ± 24.81 *μ*g/sq·cm^2^ after one hour of continuous DC iontophoresis at the end of 24 h study, respectively (Figure 2). Plot of average flux versus time (Figure 3) shows that amount of lidocaine delivered at a particular time point depends on the current applied. Modulated iontophoresis (0-1 h + 4-5 h) resulted in increased flux at each time period of current application during the duration of study, that is, 89.68 ± 35.78 *μ*g/cm^2^/h at 2 h and 64.06 ± 16.61 *μ*g/cm^2^/h at 6 h for AC iontophoresis compared to 162.96 ± 41.58 *μ*g/cm^2^/h at 2 h and 248.87 ± 116.95 *μ*g/cm^2^/h at 6 h for DC iontophoresis. The study was continued till 24 h to observe the postiontophoretic permeation and flux decreased gradually during the time period of the study. The amount of drug delivered by modulated alternating current iontophoresis for total duration of two hours at frequency of 1 kHz was found to be comparable to the drug delivered by continuous direct current iontophoresis for one hour. This may be due to the periodic polarity alteration during alternating current. It has also been reported that the driving force of the alternating current is less compared to that with application of direct current, due to periodic polarity alteration [10].

### 3.4. Drug Levels in Skin Layers following Iontophoresis Studies

**Figure 4:**
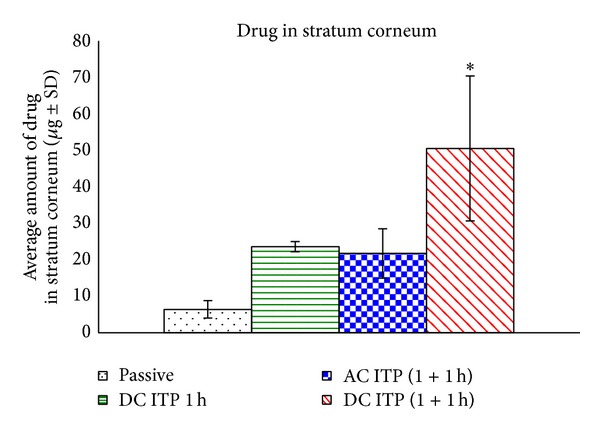
Average amount of drug in stratum corneum following iontophoresis and passive delivery. (*P < 0.05 versus passive; mean ± SD, *n* = 3).

**Figure 5:**
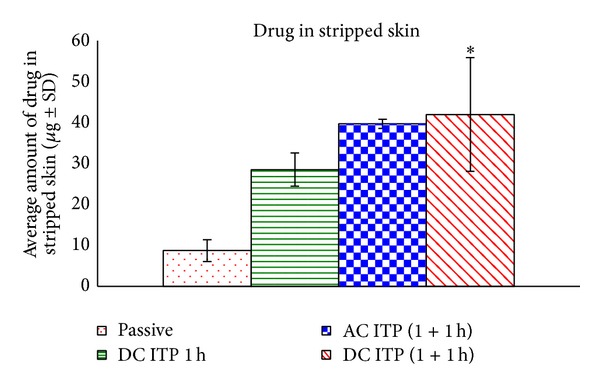
Average amount of drug in stripped skin following iontophoresis and passive delivery. (*P < 0.05 versus passive; mean ± SD, *n* = 3).

Tape stripping was done to quantify the amount of drug delivered into the stratum corneum. After tape stripping, the stripped skin was minced and extracted with 1 × PBS; pH 7.4 to quantify the drug level in stripped skin. The amount of drug delivered into the stratum corneum and stripped skin by anodal iontophoresis using alternating current and direct current iontophoresis was significantly (*P* < 0.05) higher compared to the passive diffusion as demonstrated in Figures 4 and 5. The average total amount of drug delivered into stripped skin following iontophoresis was also significantly (*P* < 0.05) higher compared to the passive delivery. The drug levels delivered into stripped skin following iontophoresis were 39.68 ± 1.11 *μ*g for AC iontophoresis and 41.98 ± 13.09 *μ*g for DC iontophoresis for two hours, which was 5-fold higher than passive delivery (8.85 ± 2.69 *μ*g). These results demonstrate the presence of rate limiting step, which restricts the movement of drug into the stripped skin. This rate-limiting step was, however, overcome by iontophoresis, as the application of current was able to propel higher level of drug into deeper skin layers as compared to passive diffusion. Quantification of lidocaine in the skin established that stratum corneum was the barrier to the delivery of this drug, as a negligible amount was detected in the skin after passive delivery.

## 4. Conclusions

Results of the iontophoretic studies demonstrated that anodal iontophoresis enhanced the delivery of lidocaine hydrochloride into and across the porcine skin. Direct current iontophoresis enhanced the permeation of lidocaine hydrochloride by twelvefold compared to passive diffusion. Direct current iontophoresis was also found to be more effective than alternating current iontophoresis in enhancing the delivery of lidocaine hydrochloride into and across the porcine skin.
